# Hypokalemia in Peritoneal Dialysis: A Systematic Review and Meta-analysis of Prevalence, Treatment, and Outcomes

**DOI:** 10.1016/j.xkme.2024.100923

**Published:** 2024-10-18

**Authors:** Changyuan Yang, Xiaoxuan Hu, Xitao Ling, Cuixia Xiao, Ruolan Duan, Jiamei Qiu, Qin Li, Xindong Qin, Jiahao Zeng, La Zhang, Haijing Hou, Yu Peng, Yuan Xu, Jingxu Su, Xusheng Liu, Bengt Lindholm, David W. Johnson, Fuhua Lu, Guobin Su

**Affiliations:** 1 State Key Laboratory of Traditional Chinese Medicine Syndrome, Department of Nephrology, The Second Affiliated Hospital of Guangzhou University of Chinese Medicine, Guangzhou, China; 2 Department of Nephrology, University Medical Center Groningen, University of Groningen, Groningen, The Netherlands; 3 Division of Renal Medicine and Baxter Novum, Department of Clinical Science, Intervention and Technology, Karolinska Institute, Stockholm, Sweden; 4 Department of Kidney and Transplant Services, Princess Alexandra Hospital, Brisbane, Australia; 5 Centre for Kidney Disease Research, University of Queensland, Brisbane, Australia; 6 Department of Medical Epidemiology and Biostatistics, Karolinska Institute, Stockholm, Sweden; 7 Nuffield Department of Population Health, University of Oxford, Oxford, UK

**Keywords:** Hypokalemia, mortality, peritoneal dialysis, potassium, prevalence

## Abstract

**Rationale & Objective:**

Hypokalemia is common and potentially life-threatening in patients undergoing peritoneal dialysis (PD). However, the current literature has produced varying results. This study aimed to evaluate the prevalence and adverse outcomes of hypokalemia and the role of potassium supplementation in patients receiving PD.

**Study Design:**

Systematic review and meta-analysis of randomized controlled trials and observational studies.

**Setting & Study Populations:**

Adults receiving maintenance PD.

**Selection Criteria for Studies:**

Studies that investigated the prevalence and adverse outcomes of hypokalemia and the effect of potassium supplementation.

**Data Extraction:**

Two independent reviewers evaluated studies for eligibility and extracted relevant data.

**Analytical Approach:**

Random effects meta-analysis was conducted to pool hazard ratios (HRs) and 95% CIs for the outcomes of interest. The certainty of findings was rated according to the Grading of Recommendations Assessment, Development and Evaluation criteria.

**Results:**

Of 3,632 reports identified, 24 studies involving 60,313 participants met the inclusion criteria. The prevalence of hypokalemia was 37.9% (95% CI, 27.2%-52.7%), 17.7% (95% CI, 12.0%-25.9%), and 4.4% (95% CI, 1.9%-10.2%) in patients with potassium level <4.0, 3.5, and 3.0 mmol/L, respectively. Hypokalemia, according to the study’s definition, was associated with increased risks of all-cause mortality (HR, 1.49; 95% CI, 1.18-1.89), cardiovascular mortality (HR, 1.50; 95% CI, 1.19-1.88), and PD-associated peritonitis (HR, 1.42; 95% CI, 1.17-1.73). These associations were consistent but with low to very low certainty. The effect of correcting hypokalemia with potassium supplementation in patients undergoing PD remains uncertain.

**Limitations:**

Heterogeneity persisted across most of the examined subgroups, and observational studies preclude causation.

**Conclusions:**

Hypokalemia is common and portends poorer survival and a higher risk of peritonitis among patients undergoing PD. Further research into the optimal prevention and treatment strategies for hypokalemia is warranted to improve outcomes.

**Registration:**

Registered at PROSPERO with registration number CRD42022358236.

## Plain-Language Summary

Hypokalemia is common and can be fatal in patients undergoing peritoneal dialysis (PD). The reported prevalence of hypokalemia in patients undergoing PD varies significantly across studies, and there is inconsistency regarding the relationship between hypokalemia and adverse outcomes. This systematic review and meta-analysis showed that hypokalemia is prevalent and associated with decreased survival rates and higher risk of peritonitis among patients undergoing PD. Standardized definitions and thresholds for hypokalemia are essential for future research in this field. The results underscore that maintaining potassium homeostasis should be a clinical priority in managing patients undergoing PD to improve patient outcomes. Further research into the optimal prevention and treatment strategies for hypokalemia is warranted to improve outcomes.

More than 3.8 million people worldwide have kidney failure and rely on some form of dialysis.^1^ Approximately 11% of patients undergoing dialysis receive peritoneal dialysis (PD).^2^ Potassium is an important body electrolyte. Because serum potassium levels are maintained in the normal range primarily by the kidneys, patients with kidney failure are prone to have dyskalemia.^3^, ^4^, ^5^ In contrast to patients receiving hemodialysis or those with kidney failure, hypokalemia is more prevalent in patients undergoing PD, ranging from 10%-76%.^6^, ^7^, ^8^ The reasons for the varying prevalence of hypokalemia in patients receiving PD may in part be because of the different definitions and thresholds by which studies report hypokalemia, differences in dietary intakes of potassium, PD prescription, and the use of oral potassium supplementation in different regions.^9^^,^^10^ Therefore, uncertainty persists regarding the prevalence of hypokalemia in patients undergoing PD, which in turn limits a clear understanding of the actual extent of hypokalemia’s impact on these patients.

Some epidemiologic studies have explored the association between hypokalemia and adverse outcomes including all-cause mortality,^6^^,^^8^^,^^11^ cardiovascular mortality,^11^, ^12^, ^13^ and peritonitis^8^^,^^14^ among patients undergoing PD, but with inconsistent results. In addition, although hypokalemia may be associated with several adverse outcomes as mentioned above, there are inconsistent results about the association of potassium supplementation with survival in patients with PD.^15^^,^^16^

Therefore, the objective of this study was to offer a comprehensive overview of the epidemiology of hypokalemia to date, encompassing a spectrum of hypokalemia thresholds across various health care settings and continents in patients receiving PD. Furthermore, we aimed to investigate the adverse outcomes linked with hypokalemia and explore the role of potassium supplementation in this population.

## Methods

### Study Registration

This study was conducted according to the Preferred Reporting Items for Systematic Reviews and Meta-analyses guidelines (Table S1). The study protocol was prospectively registered on PROSPERO (CRD42022358236).^17^

### Data Sources and Searches

We searched MEDLINE, Embase, and Web of Science without language restrictions until June 2024 (Table S2). Author pairs completed the title and abstract screening and full-text screening, and they manually searched the reference lists of eligible studies and reviews to identify other potentially relevant studies by snowballing.

### Inclusion and Exclusion Criteria

A study was considered eligible according to the following criteria: (1) randomized controlled trial (RCT), cohort study, or cross-sectional study; (2) adults receiving maintenance PD; and (3) report of a specific threshold to define hypokalemia. If a study did not provide a definition or threshold for hypokalemia, it was included if the authors reported the proportion of patients with serum potassium levels <4 mmol/L; and 4) reported ≥1 outcome of interest. Moreover, studies with limited sample size (<100) were excluded to mitigate potential imprecision.

### Exposure

We assessed a wide range of hypokalemia severity with different cutoffs including serum potassium levels of <3.0, <3.5, and <4.0 mmol/L, regardless of whether it was based on a single measurement or time-averaged values of multiple measurements of serum potassium levels.

### Outcomes of Interest

The outcomes of interest were the prevalence of hypokalemia and the association of hypokalemia with adverse events including all-cause mortality, cardiovascular mortality, infection-related mortality, and PD-associated peritonitis. A detailed definition of the outcomes is provided in Table S3. The safety and outcomes of pharmacologic or dietary interventions aimed at correcting hypokalemia in patients receiving PD were also assessed.

### Data Extraction

Two reviewers (C.Y. and X.H.) independently extracted the data from the included studies using standardized data extraction forms created in Microsoft Excel (Table S3). When questions arose concerning a study’s eligibility and missing data, we contacted corresponding authors to request this information (if applicable).

### Quality Assessment

Two review authors (C.Y. and X. Ling) independently evaluated the risk of bias for each study. For cohort and case-control studies, the risk of bias was assessed using the Newcastle–Ottawa Scale tool.^18^ Study quality scores were defined arbitrarily as poor (0-3), fair (4-6), or good (7-9). Cross-sectional studies were evaluated for bias using the Joanna Briggs Institute Critical Appraisal Checklist for Prevalence Studies.^19^ The risk of bias of RCTs was assessed using Cochrane’s Risk of Bias assessment tool.^20^ Any disagreement was assessed by a third author (G.S.). We applied the Grading of Recommendations Assessment, Development and Evaluation methodology criteria to rate the quality of evidence (low, moderate, or high) for each outcome.

### Data Synthesis and Statistical Analysis

All prevalence data were log-transformed, and pooled prevalence (with 95% confidence interval [CI]) was recalculated. The overall association estimates for all outcomes of interest were expressed as hazard ratios (HRs) or odds ratios with 95% CI and the most adjusted-for cofounders were used. Given the observational design and highly varied sample sizes of the primary studies, we used the random effects model (DerSimonian and Laird) for all meta-analyses using the “metafor” package in R. In instances in which comparisons of different levels of potassium were reported or multiple publications covered the same study population, the most extreme comparison and the one with longer follow-up were used.

*I*^*2*^ was used to measure heterogeneity across studies, which was categorized as low (0%-50%), moderate (51%-75%), or high (>75%). The leave-one-study method was used to examine whether the overall estimate was influenced by the substantial heterogeneity observed.^21^ Interrater agreement for the final selection of the articles was evaluated by calculating Cohen’s κ coefficient using the “psych” package in R. Furthermore, we performed additional empirical Bayes meta-regression modeling of studies addressing heterogeneity. Subgroup analyses were conducted according to the threshold of hypokalemia (<4.0, <3.5, and <3.0 mmol/L); definition of hypokalemia (single measurement, average of multiple measurements, or unclear); continents (Asia vs non-Asia); different countries or regions; and sample size (above vs below the median). In analyses that included ≥10 studies, when the primary outcome showed low or moderate heterogeneity, we constructed funnel plots to check for effects that may represent publication, selection, or reporting bias. Additionally, funnel plot asymmetry was further confirmed with Egger’s test.^22^ A two-tailed *P* values < 0.05 were considered statistically significant. All statistical analyses were conducted using R (version 4.1.1; R Foundation for Statistical Computing).

## Results

**Figure 1:**
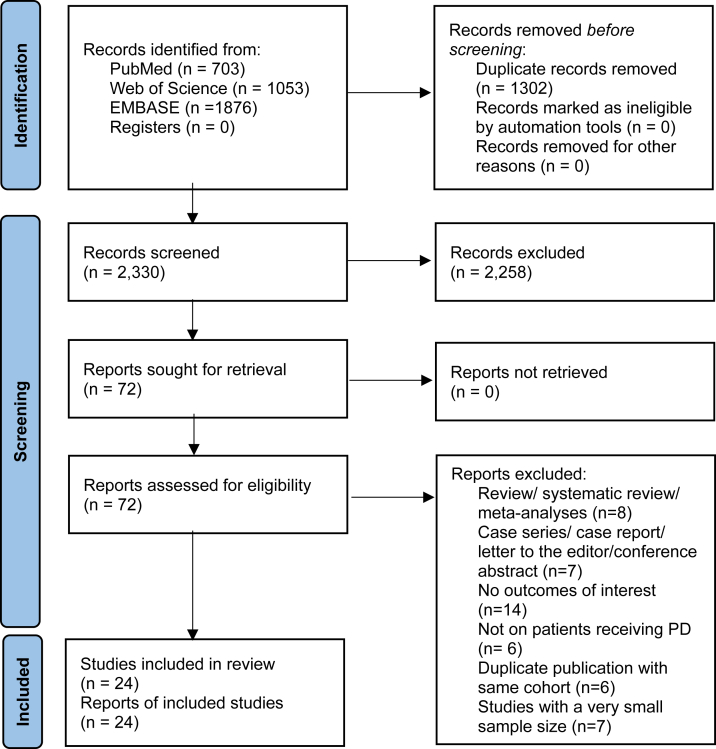
Flowchart showing the study selection process. Abbreviations: PD, peritoneal dialysis.

The electronic search retrieved 3,632 citations, of which 24 studies encompassing 60,313 participants met the inclusion criteria (Fig 1).^6^, ^7^, ^8^^,^^11^, ^12^, ^13^, ^14^, ^15^, ^16^^,^^23^, ^24^, ^25^, ^26^, ^27^, ^28^, ^29^, ^30^, ^31^, ^32^, ^33^, ^34^, ^35^, ^36^, ^37^ The interrater agreement κ coefficient for the final selection of the articles was 0.84 (95% CI, 0.72-0.96). The characteristics of the included studies are reported in Table 1. The sample size of each study ranged from 110-17,664. The design of eligible studies included: RCT (n = 1),^15^ cohort study (n = 19),^6^^,^^8^^,^^11^, ^12^, ^13^, ^14^^,^^16^^,^^23^^,^^24^^,^^26^, ^27^, ^28^, ^29^, ^30^, ^31^^,^^33^^,^^34^^,^^36^^,^^37^ and cross-sectional study (n = 4).^7^^,^^25^^,^^32^^,^^35^ Over half of the studies used time-averaged or multiple measurements of serum potassium levels to define hypokalemia (n = 14),^6^, ^7^, ^8^^,^^12^^,^^13^^,^^15^^,^^16^^,^^24^^,^^25^^,^^27^^,^^28^^,^^30^^,^^32^^,^^35^ while 6 studies^14^^,^^26^^,^^29^^,^^34^^,^^36^^,^^37^ only employed a single serum potassium measurement. Four studies^11^^,^^23^^,^^31^^,^^33^ did not explicitly describe how hypokalemia was defined. The most-used threshold of hypokalemia was serum potassium concentrations of <3.5 mmol/L (n = 21). Fifteen studies investigated the relationship between hypokalemia and adverse outcomes, including all-cause mortality (n = 10),^6^^,^^8^^,^^11^, ^12^, ^13^^,^^16^^,^^24^^,^^28^^,^^30^^,^^34^ cardiovascular mortality (n = 7),^6^^,^^11^, ^12^, ^13^^,^^16^^,^^30^^,^^34^ PD-associated peritonitis (n = 6),^8^^,^^14^^,^^26^^,^^27^^,^^33^^,^^36^ and infection-related mortality (n = 2)^6^^,^^30^ (Table S4). Almost all studies investigated a single outcome, either mortality or peritonitis, except for 1 study, which reported on both.^8^ Table S5 provides an overview of the confounders adjusted for the included studies.

**Table 1:** Baseline Characteristics of the Included Studies

Study (Y)	Country	Study Design	Sample Size	Age, y, Mean ± SD	Male (%)	Potassium Measurement	
						Times of Measurements	Definition
**Huang** et al^11^ (**2023**)	China	Prospective cohort study	6,605	51.7 ± 14.8	55.3%	Unclear	Threshold only^a^
**Pan** et al^37^ (**2023**)	China	Retrospective cohort study	880	57.7 ± 14.8	49.3%	Single	Once less than normal range
**Pichitporn** et al^15^ (**2022**)	Thailand	Randomized controlled trial	167	55.0 ± 11.8	41.9%	Multiple	Spot serum potassium or average serum potassium ≥3 measurements
**Huo** et al^27^ (**2022**)	China	Retrospective cohort study	1,633	46.6 ± 13.8	54.0%	Multiple	Total number of months
**Liu** et al^26^ (**2021**)	China	Retrospective cohort study	602	45.0 ± 15.1	58.2%	Single	Once less than normal range
**Davies** et al^8^ (**2021**)	Australia, Canada, Japan, New Zealand, Thailand, United Kingdom, United States	Retrospective cohort study	7,596	59.2 ± 14.9	NA	Multiple	Monthly-averaged (4 mo) or number of months
**Tangjittrong**^33^ (**2021**)	Thailand	Retrospective cohort study	411	57.5 ± 13.8	55.2%	Unclear	Threshold only
**Tatiyanupanwong** et al^36^ (**2020**)	Thailand	Retrospective cohort study	947	56.1 ± 12.8	46.9%	Single	Once less than normal range
**Goncalves** et al^7^ (**2020**)	Brazil	Cross-sectional study	146	55 ± 18	52.7%	Multiple	Number of episodes of hypokalemia
**Hamad** et al^32^ (**2019**)	Qatar	Cross-sectional study	143	NA	NA	Multiple	Persistent hypokalemia or a continuous need for potassium replacement ≥3 mo despite regular management
**Eriguchi** et al^12^ (**2019**)	United States	Retrospective cohort study	17,664	56 ± 16	56%	Multiple	Monthly-averaged
**Lee** et al^24^ (**2017**)	Korea	Prospective cohort study	3,230	54 (45-64)^b^	56.5%	Multiple	The 3-mo visit, and subsequently every 6 mo until year 5
**Zhang** et al^16^ (**2016**)	China	Retrospective cohort study	676	53.7 ± 11.3	58.7%	Multiple	Monthly-averaged (3 consecutive months)
**Liu** et al^35^ (**2016**)	China	Cross-sectional study	319	50.8 ± 14.7	52.7%	Multiple	Monthly-averaged (first 3 mo after PD initiation)
**Ribeiro** et al^30^ (**2015**)	Brazil	Prospective cohort study	5,408	59.3 ± 15.9	48%	Multiple	Monthly-averaged
**Li** et al^13^ (**2015**)	China	Retrospective cohort study	357	50.3 ± 15.6	54.3%	Multiple	Time-averaged (24 calendar quarters)
**Xu** et al^34^ (**2014**)	China	Retrospective cohort study	886	48.56 ± 15.4	57.1%	Single	Once less than normal range
**Fan** et al^14^ (**2014**)	China	Retrospective cohort study	1,086	NA	NA	Single	Once less than normal range
**Vavruk** et al^25^ (**2012**)	Brazil	Cross-sectional study	110	62 (22-91)^b^	46.4%	Multiple	6 consecutive measurements
**Torlén** et al^6^ (**2012**)	United States	Retrospective cohort study	10,468	56 ± 16	53%	Multiple	Up to 20 calendar quarters
**Liawnoraset**^31^ (**2011)**	Thailand	Retrospective cohort study	318	49.9 ± 14.3	50.9%	Unclear	Threshold only
**Chuang** et al^23^ (**2009**)	Taiwan	Retrospective cohort study	140	47.8 ± 16.0	39.3%	Unclear	Threshold only
**Yi** et al^29^ (**2009**)	South Korea	Retrospective cohort study	255	55.2 ± 12.5	48.6%	Single	Once less than normal range
**Szeto** et al^28^ (**2005**)	China	Retrospective cohort study	266	51.2 ± 15.0	50.8%	Multiple	3 consecutive monthly measurements

Abbreviations: NA, not available; PD, peritoneal dialysis; SD, standard deviation.

a This study only defined the threshold (such as potassium below 4.0, 3.5, or 3.0 mmol/L) for hypokalemia.

b Presented as median (range).

### Quality Assessment

In general, the quality of the included cohort studies (n = 19) was moderate according to the Newcastle–Ottawa Scale criteria (4 studies^23^^,^^31^^,^^33^^,^^36^ with fair quality and 15 studies with good quality^6^^,^^8^^,^^11^, ^12^, ^13^, ^14^^,^^16^^,^^24^^,^^26^, ^27^, ^28^, ^29^, ^30^^,^^34^^,^^37^) (Table S6). All studies had a low risk of bias for the representativeness of the sample and assessment of outcome. The common item underscored in this scale was an unclear definition of hypokalemia (n = 4).^11^^,^^23^^,^^31^^,^^33^ With respect to follow up, 89% of studies had a low risk of bias, and bias was judged high risk in 2 studies^33^^,^^36^ because of very short follow-up periods (<2 years). In addition, one RCT^15^ scored 6 of 8 stars based on Cochrane’s Risk of Bias assessment tool (Table S7).

The certainty (Grading of Recommendations Assessment, Development and Evaluation) of the evidence for each outcome meta-analyzed is detailed in Table S8. The 4 included cross-sectional studies^7^^,^^25^^,^^32^^,^^35^ scored 6-8 of 9 based on the Joanna Briggs Institute appraisal checklist (Table S9). Although all studies met the criteria regarding sampling frame, subject and setting, valid measures, and standardized measures, no study met the criteria regarding appropriate statistical analysis, mainly because of missing CIs of hypokalemia prevalence.

### Prevalence of Hypokalemia

**Figure 2:**
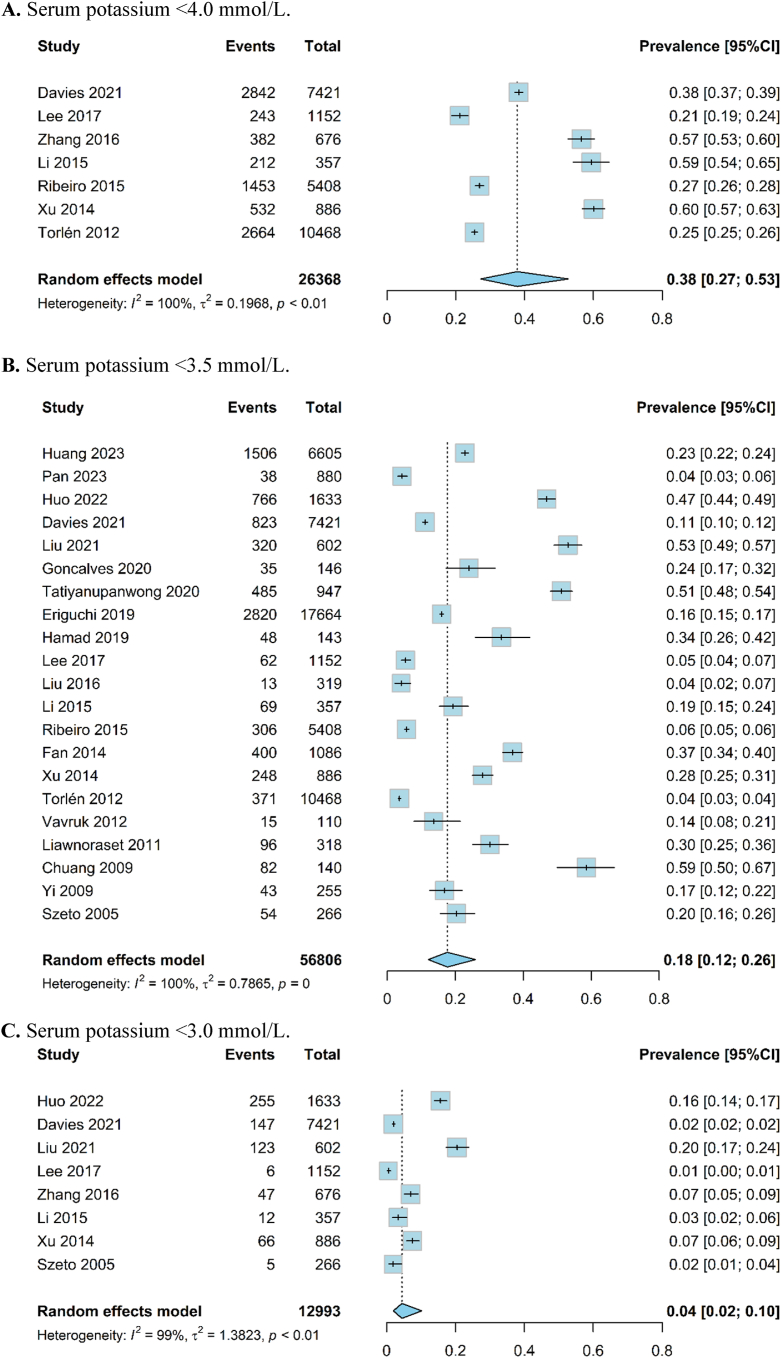
Meta-analysis of the prevalence of hypokalemia with a serum potassium threshold below (A) 4.0 mmol/L (n = 7); (B) 3.5 mmol/L (n = 21); and (C) 3.0 mmol/L (n = 8).

Twenty-two studies^6^, ^7^, ^8^^,^^11^, ^12^, ^13^, ^14^^,^^16^^,^^23^, ^24^, ^25^, ^26^, ^27^, ^28^, ^29^, ^30^, ^31^, ^32^^,^^34^, ^35^, ^36^, ^37^ involving 57,482 adult participants reported prevalence data from 12 countries or regions. The overall pooled prevalence for hypokalemia was 37.9% (95% CI, 27.2%-52.7%; *I*^*2*^ = 100%; n = 7; evidence quality: very low), 17.7% (95% CI: 12.0%-25.9%; *I*^*2*^ = 100%; n = 21; evidence quality: very low), and 4.4% (95% CI: 1.9%-10.2%; *I*^*2*^ = 99%; n = 8; evidence quality: very low) for patients with potassium level <4.0, 3.5 and 3.0 mmol/L, respectively (Fig 2). Subgroup analyses showed a higher prevalence of hypokalemia in studies from Asia, studies with hypokalemia defined by a single measurement, and studies with small sample sizes (Table 2).

**Table 2:** Pooled Prevalence of Hypokalemia Among Adult Patients Undergoing Peritoneal Dialysis Across All Studies and Subgroup Analyses

Characteristics	Serum Potassium (mmol/L)								
	<4.0 mmol/L			<3.5 mmol/L			<3.0 mmol/L		
	Studies (n)	% (95% CI)	*I*^*2*^ (%)	Studies (n)	% (95% CI)	*I*^*2*^ (%)	Studies (n)	% (95% CI)	*I*^*2*^ (%)
**Overall**	7	37.9% (27.2%-52.7%)	100%	21	17.7% (12.0%-25.9%)	100%	8	4.4% (1.9%-10.2%)	99%
**Subgroup analyses**									
**Continent**^a^									
**Asia**	6	47.5% (32.6%-69.1%)	99%	17	22.2% (14.8%-33.4%)	99%	9	5.2% (2.4%-11.3%)	97%
**Non-Asia**	6	28.7% (24.3%-34.0%)	98%	9	7.7% (4.9%-12.1%)	99%	4	0.2% (0.02%-1.1%)	76%
**Countries or regions**^b^									
**China**	3	58.7% (56.4%-61.2%)	1%	9	19.9% (10.9%-36.2%)	99%	6	7.3% (3.7%-14.5%)	96%
**Thailand**	1	76.0% (73.1%-79.0%)	NA	3	42.1% (30.8%-57.5%)	94%	1	16.0% (13.7%-18.7%)	NA
**Japan**	1	35.0% (31.8%-38.4%)	NA	1	12.0% (9.9%-14.4%)	NA	1	2.0% (1.2%-3.2%)	NA
**Korea**	1	21.1% (18.9%-23.6%)	NA	2	9.5% (3.1%-29.1%)	97%	1	0.5% (0.2%-1.2%)	NA
**United States**	2	29.8% (21.8%-40.8%)	99%	3	7.0% (3.0%-16.6%)	100%	1	0.01% (0%-0.2%)	NA
**Brazil**	1	26.9% (25.7%-28.1%)	NA	3	12.1% (5.2%-28.3%)	98%	NA	NA	NA
**Canada**	1	39.0% (35.8%-42.3%)	NA	1	9.0% (7.3%-11.1%)	NA	1	1.1% (0.6%-2.0%)	NA
**United Kingdom**	1	22.9% (18.7%-27.8%)	NA	1	3.1% (1.7%-5.6%)	NA	1	0	NA
**Australia/New Zealand**	1	25.1% (21.5%-29.0%)	NA	1	5.1% (3.5%-7.4%)	NA	1	0	NA
**Potassium measurement methods**									
**Multiple**	6	35.1% (24.8%-49.6%)	100%	12	12.7% (7.9%-20.4%)	100%	6	3.1% (1.2%-7.9%)	99%
**Single**	1	60.1% (56.9%-63.4%)	NA	6	24.5% (11.6%-51.8%)	99%	2	12.4% (4.6%-33.3%)	98%
**Unclear**	0	NA	NA	3	34.2% (19.7%-59.3%)	99%	0	NA	NA
**Sample size**^c^									
**<** **Median**	3	58.7% (56.4%-61.2%)	1%	10	22.7% (14.4%-35.7%)	97%	4	5.8% (2.1%-15.8%)	97%
**≥ Median**	4	27.4% (21.4%-34.9%)	99%	11	14.2% (7.9%-25.6%)	100%	4	3.4% (0.8%-14.5%)	99%

Abbreviations: NA, not applicable.

a Davies et al^8^ included multiple (n = 6) countries or regions, including Australia/New Zealand, Canada, Japan, Thailand, United Kingdom, and United States. Each country was individually considered in continent and country subgroup analyses.

b Countries where the prevalence of hypokalemia was reported ≥3 times are shown in this table.

c Using the median of sample size from the included studies of different potassium levels as the cutoff point.

### Hypokalemia and Adverse Outcomes

Most studies investigating adverse outcomes defined hypokalemia as serum potassium level <3.5 mmol/L, which was used in most all-cause mortality studies (40%), cardiovascular mortality studies (43%), infection-related mortality studies (100%), and PD-associated peritonitis studies (100%) (Table S4).

#### All-Cause Mortality

**Figure 3:**
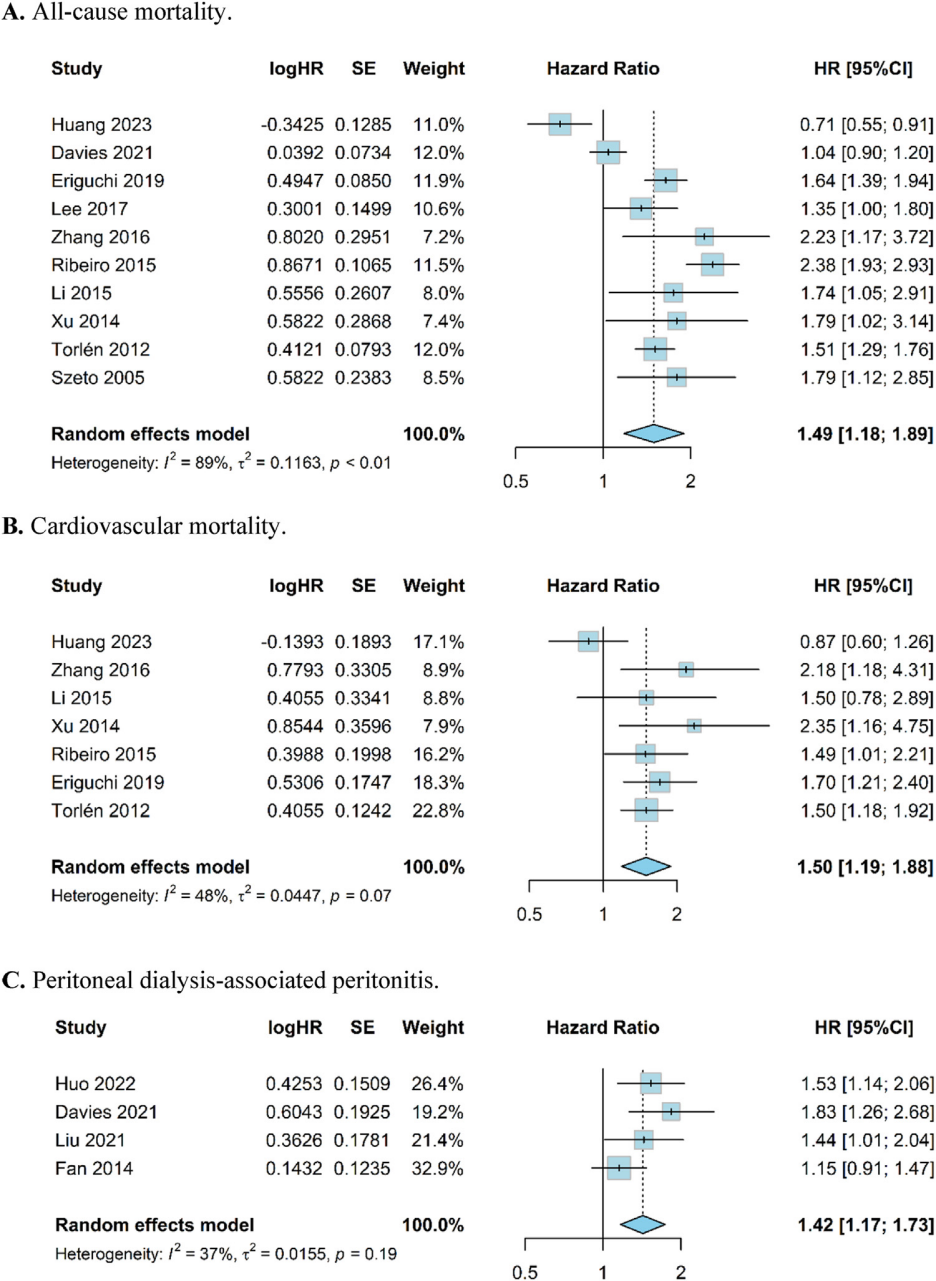
Forest plot of association between hypokalemia and clinical outcomes in patients receiving peritoneal dialysis. (A) Hypokalemia (by study definition) and all-cause mortality (n = 10). (B) Hypokalemia (by study definition) and cardiovascular mortality (n = 7). (C) Hypokalemia (defined by serum potassium level <3.5 mmol/L) and peritoneal dialysis-associated peritonitis (n = 4).

Hypokalemia, according to the study definition, was associated with a 49% increased risk of all-cause mortality among patients with PD (HR, 1.49; 95% CI, 1.18-1.89; *I*^*2*^ = 89%; 53,156 participants; evidence quality: low) than those without hypokalemia (Fig 3).

#### Cardiovascular and Infection-Related Mortality

Individuals with hypokalemia by study definition exhibited a 50% higher risk of cardiovascular mortality (HR, 1.50; 95% CI, 1.19-1.88; *I*^*2*^ = 48%; 31,996 participants; evidence quality: moderate) than those without hypokalemia (Fig 3). Only 2 studies explored the relationship between hypokalemia and infection-related mortality, with both highlighting that hypokalemia (<3.5 mmol/L) was a risk factor for infection-related mortality (Table S4).

#### PD-Associated Peritonitis

The pooled risk for PD-associated peritonitis was 42% higher in those with hypokalemia, defined by serum potassium level of <3.5 mmol/L (HR, 1.42; 95% CI, 1.17-1.73; *I*^*2*^ = 37%; 10,917 participants; evidence quality: low) from 4 studies (Fig 3). Two studies were excluded from the meta-analysis of PD-associated peritonitis because they only reported odds ratio data (Table S4).^33^^,^^36^

#### Subgroup Analyses

Subgroup analyses categorized by different potassium levels consistently demonstrated an association between all-cause mortality and hypokalemia <3.5 mmol/L and <3.0 mmol/L, but not with the cutoff of <4.0 mmol/L (Fig S1). A similar association was also observed between hypokalemia and cardiovascular mortality (Fig S2).

### Potassium Supplementation and Outcomes

Only 2 studies investigated potassium supplementation in patients receiving PD. An RCT^15^ found that compared with reactive potassium supplementation when serum potassium levels fell <3.5 mmol/L, protocol-based oral potassium treatment to maintain serum potassium concentration in the range of 4-5 mmol/L may reduce the risk of peritonitis in patients receiving PD (HR, 0.47; 95% CI, 0.24-0.93), but not with all-cause and cardiovascular mortality. Similarly, a retrospective cohort study^16^ involving 676 patients receiving PD indicated that the use of oral potassium supplementation was not associated with a lower risk of all-cause or cardiovascular mortality compared with no oral potassium supplementation. However, this study did not provide information regarding the use of oral potassium supplementation (eg, dosage and duration).

### Sensitivity Analyses and Meta-regression

The leave-one-study sensitivity analysis demonstrated that the association between hypokalemia and all-cause mortality remained consistent (Fig S3). Meta-regression analyses indicated that studies with larger sample sizes showed lower HRs of outcomes when compared with their counterparts, although the results did not reach statistical significance (Table S10).

### Publication Bias

The Egger’s test analysis showed no indication of publication bias for meta-analysis of the prevalence of hypokalemia (*P* = 0.65) and its association with all-cause mortality (*P* = 0.55), as shown in Figs S4-S5.

## Discussion

In this systematic review and meta-analysis of 24 studies involving 60,313 participants, we found that hypokalemia was common and served as an important risk factor for all-cause mortality, cardiovascular mortality, and PD-associated peritonitis in patients receiving PD. This study adds to the body of knowledge that hypokalemia is a severe problem among patients undergoing PD. The study underscores differences in reported definitions or thresholds of hypokalemia and identifies the critical need for a unified approach to address hypokalemia and its consequences in patients receiving PD.

This study shows a significant burden of hypokalemia among patients receiving PD, with the overall pooled prevalence estimates ranging from 4.4% (<3.0 mmol/L) to 37.9% (<4.0 mmol/L). This wide range may be attributed to several factors. First, differences existed between different regions or PD centers in dietary patterns, medication usage (such as renin-angiotensin-aldosterone system inhibitors or potassium supplementation), body composition, and PD prescriptions.^38^^,^^39^ Further subgroup analyses indicated that the pooled prevalence of hypokalemia in Asia (ie, mainly East and Southeast Asia) is 1.5-3 times higher than that in Western countries when using cutoffs of 4.0 and 3.5 mmol/L and over 25 times higher when using a cutoff of 3.0 mmol/L. Thailand reached the highest prevalence of hypokalemia in patients receiving PD among the included countries, with rates of 76.0% and 42.1% for cutoffs of 4.0 and 3.5 mmol/L, respectively. Hypokalemia is considered a surrogate for protein-energy wasting,^8^^,^^25^ and the higher prevalence of protein-energy wasting in patients with chronic kidney disease (CKD) in Southeast Asia may explain this high prevalence.^40^ Second, despite the establishment of blood potassium thresholds, there was substantial variation in the definition of hypokalemia across studies. Our further subgroup analysis showed that the prevalence of hypokalemia defined based on multiple measurements is evidently lower than that based on a single measurement across all thresholds. Additionally, it should be noted that although more than half of the studies included in this research defined hypokalemia based on the average of multiple measurements or the frequency to meet the cutoff, there was considerable variation in the observation time frame and the frequency of tests across different studies, which also contributed to the variation of hypokalemia.

Our study demonstrates that hypokalemia was a significant risk factor for all-cause and cardiovascular mortality in patients receiving PD. The association of hypokalemia with mortality likely involves several mechanisms. First, hypokalemia can precipitate life-threatening cardiac arrhythmias and impair myocardial contractility.^41^^,^^42^ Second, hypokalemia may indicate a catabolic state and malnutrition, which are independent predictors of mortality in dialysis patients.^43^ Several previous systematic reviews have established the relationship between hypokalemia and mortality in patients with CKD but did not distinguish between non–dialysis-dependent and dialysis-dependent CKD patients.^44^^,^^45^ They overlooked the unique characteristics of patients receiving PD (significantly higher prevalence of hypokalemia than other patients receiving hemodialysis^6^) and did not specifically investigate the correlation between hypokalemia and cause-specific mortality in patients receiving PD. Furthermore, although a recent systematic review^46^ identified that levels of serum potassium may be a risk factor for mortality in patients receiving PD, it failed to associate low serum potassium levels with all-cause mortality (HR, 1.25; 95% CI, 0.97-1.60; n = 3) or cardiovascular mortality (HR, 1.46; 95% CI, 0.67-3.16, n = 2). This may be attributed to incomplete data retrieval and unclear definitions or thresholds of hypokalemia. Our study confirms and extends previous evidence linking hypokalemia to these outcomes in patients receiving PD.

We found compelling evidence that hypokalemia is associated with an increased risk of PD-associated peritonitis. Possible explanations are as follows. First, individuals with hypokalemia tend to have poorer nutritional status, higher levels of inflammatory markers, and lower albumin concentrations.^47^ This may potentially render them more susceptible to the onset of peritonitis.^47^ Second, hypokalemia slows gut motility and this may lead to an increased risk of translocation of such bacteria across the bowel wall, leading to peritonitis.^23^ A systematic review published in 2017 could not draw a clear conclusion regarding the association between hypokalemia and peritonitis in patients receiving PD because of lack of power.^48^ However, a recent international investigation from the Peritoneal Dialysis Outcomes and Practice Patterns Study^8^ involving 7,421 patients from 7 countries showed an association between hypokalemia and peritonitis.

The optimal management of hypokalemia and the effect of correction of hypokalemia through potassium supplementation on outcomes, such as mortality and peritonitis risk, remain uncertain. After comprehensive literature retrieval, we only identified 1 RCT that investigated the relationship between potassium supplementation and adverse outcomes in patients receiving PD.^15^ However, it was unable to demonstrate survival benefits. This may have been related to the challenges in maintaining stable potassium levels with oral potassium supplementation or the relatively short-term duration and probably insufficient intervention length to observe long-term survival benefits. Additionally, recent evidence also highlights the potential effect of potassium-sparing diuretics in maintaining normal serum potassium levels in patients receiving PD.^49^ Therefore, further research is warranted to clarify the efficacy and safety of potassium supplementation and potassium-sparing diuretics in PD patients with hypokalemia.

Our study has strengths including comprehensive searches covering a large number of health indicators such as prevalence, all-cause and cause-specific mortality, and peritonitis; extensive sensitivity, subgroup, and meta-regression analyses; and exploration of the role of potassium supplementation intervention in mitigating adverse outcomes in patients receiving PD. However, the following limitations should be noted when interpreting the results. First, we identified significant heterogeneity in the overall estimates. Despite conducting several subgroup analyses to mitigate heterogeneity, it was challenging to perform all these analyses owing to the limited data available. Furthermore, limited data also hindered us from further exploring the relationship between the dose of potassium and outcomes. Second, the potential impact of reporting bias should be noted. Despite our thorough literature search, there was a potential for publication bias in studies in which positive or statistically significant results may have been selectively published, possibly overestimating hypokalemia effects. Third, we included the estimates with the most extreme comparison when different levels of potassium were reported within the same study. This may lead to overestimation of the effect of hypokalemia to some degree, as the most extreme comparison with the reference level was not always selected. Fourth, it is worth noting that most of the studies included in this systematic review originated from Asia (ie, China and Thailand). Interpretation and generalization of our study to other settings or regions should be made with caution. Finally, considering that our study was mostly based on observational studies, causality and residual or unmeasured confounding could not be eliminated.

In conclusion, this study indicates that hypokalemia is common and portends poorer survival and a higher risk of peritonitis among patients receiving PD. These results underline that maintaining potassium homeostasis should be a clinical priority in managing patients receiving PD to improve patient outcomes. Standardized definitions for hypokalemia are essential for future research in this field.

## References

[1] Teitelbaum I. (2021). Peritoneal dialysis. N Engl J Med.

[2] Cho Y., Bello A.K., Levin A. (2021). Peritoneal dialysis use and practice patterns: an international survey study. Am J Kidney Dis.

[3] Kovesdy C.P., Appel L.J., Grams M.E. (2017). Potassium homeostasis in health and disease: a scientific workshop cosponsored by the National Kidney Foundation and the American Society of Hypertension. Am J Kidney Dis.

[4] Gilligan S., Raphael K.L. (2017). Hyperkalemia and hypokalemia in CKD: prevalence, risk factors, and clinical outcomes. Adv Chronic Kidney Dis.

[5] Elliott A.B., Soliman K.M.M., Ullian M.E. (2022). Hyperkalemia in chronic peritoneal dialysis patients. Ren Fail.

[6] Torlén K., Kalantar-Zadeh K., Molnar M.Z., Vashistha T., Mehrotra R. (2012). Serum potassium and cause-specific mortality in a large peritoneal dialysis cohort. Clin J Am Soc Nephrol.

[7] Goncalves F.A., de Jesus J.S., Cordeiro L. (2020). Hypokalemia and hyperkalemia in patients on peritoneal dialysis: incidence and associated factors. Int Urol Nephrol.

[8] Davies S.J., Zhao J., Morgenstern H. (2021). Low serum potassium levels and clinical outcomes in peritoneal dialysis—international results from PDOPPS. Kidney Int Rep.

[9] Tziviskou E., Musso C., Bellizzi V. (2003). Prevalence and pathogenesis of hypokalemia in patients on chronic peritoneal dialysis: one center’s experience and review of the literature. Int Urol Nephrol.

[10] Virojanawat M., Puapatanakul P., Chuengsaman P. (2021). Hypokalemia in peritoneal dialysis patients in Thailand: the pivotal role of low potassium intake. Int Urol Nephrol.

[11] Huang N., Liu Y., Ai Z. (2023). Mediation of serum albumin in the association of serum potassium with mortality in Chinese dialysis patients: a prospective cohort study. Chin Med J (Engl).

[12] Eriguchi R., Obi Y., Soohoo M. (2019). Racial and ethnic differences in mortality associated with serum potassium in incident peritoneal dialysis patients. Am J Nephrol.

[13] Li S.H., Xie J.T., Long H.B. (2015). Time-averaged serum potassium levels and its fluctuation associate with 5-year survival of peritoneal dialysis patients: two-center based study. Sci Rep.

[14] Fan X., Huang R., Wang J., Jia Z. (2014). Risk factors for the first episode of peritonitis in Southern Chinese continuous ambulatory peritoneal dialysis patients. PLoS ONE.

[15] Pichitporn W., Kanjanabuch T., Phannajit J. (2022). Efficacy of potassium supplementation in hypokalemic patients receiving peritoneal dialysis: a randomized controlled trial. Am J Kidney Dis.

[16] Zhang Y.F., Wang Q., Su Y.Y. (2016). Potassium supplementation and long-term outcomes in chronic peritoneal dialysis patients with end-stage renal disease: a propensity score matching study. Ren Fail.

[17] Moher D., Liberati A., Tetzlaff J., Altman D.G., PRISMA Group (2009). Preferred Reporting Items for Systematic Reviews and Meta-Analyses: the PRISMA statement. Ann Intern Med.

[18] Wells G.A., Shea B., O’Connell D. (2021). The Newcastle-Ottawa Scale (NOS) for Assessing the Quality of Nonrandomised Studies in Meta-Analyses. Ottawa Hospital Research Institute. https://www.ohri.ca/programs/clinical_epidemiology/oxford.asp.

[19] Munn Z., Moola S., Riitano D., Lisy K. (2014). The development of a critical appraisal tool for use in systematic reviews addressing questions of prevalence. Int J Health Policy Manag.

[20] Sterne J.A.C., Savović J., Page M.J. (2019). RoB 2: a revised tool for assessing risk of bias in randomised trials. BMJ.

[21] Viechtbauer W., Cheung M.W.L. (2010). Outlier and influence diagnostics for meta-analysis. Res Synth Methods.

[22] Egger M., Davey Smith G., Schneider M., Minder C. (1997). Bias in meta-analysis detected by a simple, graphical test. BMJ.

[23] Chuang Y.W., Shu K.H., Yu T.M., Cheng C.H., Chen C.H. (2009). Hypokalaemia: an independent risk factor of enterobacteriaceae peritonitis in CAPD patients. Nephrol Dial Transplant.

[24] Lee S., Kang E., Yoo K.D. (2017). Lower serum potassium associated with increased mortality in dialysis patients: A nationwide prospective observational cohort study in Korea. PLOS ONE.

[25] Vavruk A.M., Martins C., Nascimento M.M., Hayashi S.Y., Riella M.C. (2012). Association between hypokalemia, malnutrition and mortality in peritoneal dialysis patients. J Bras Nefrol.

[26] Liu D., Lin Y., Gong N. (2021). Degree and duration of hypokalemia associated with peritonitis in patients undergoing peritoneal dialysis. Int J Clin Pract.

[27] Huo Z., Zhuo Q., Zhong S. (2022). Hypokalemia duration in the first year associated with subsequent peritoneal dialysis-associated peritonitis: a multicenter retrospective cohort study. J Clin Med.

[28] Szeto C.C., Chow K.M., Kwan B.C.H. (2005). Hypokalemia in Chinese peritoneal dialysis patients: prevalence and prognostic implication. Am J Kidney Dis.

[29] Yi J.H., Park J.I., Choi H.Y., Lee H.Y., Han S.W., Kim H.J. (2009). Icodextrin improves the serum potassium profile with the enhancement of nutritional status in continuous ambulatory peritoneal dialysis patients. Electrolyte Blood Press.

[30] Ribeiro S.C., Figueiredo A.E., Barretti P., Pecoits-Filho R., de Moraes T.P. (2015). all centers that contributed to the BRAZPD II study. Low serum potassium levels increase the infectious-caused mortality in peritoneal dialysis patients: a propensity-matched score study. PLOS ONE.

[31] Liawnoraset W. (2011). Prevalence and factors affecting peritonitis in CAPD patients in Maharat Nakhon Ratchasima Hospital under universal coverage scheme during 2008-2010: a three-year experience. J Med Assoc Thai.

[32] Hamad A., Hussain M.E., Elsanousi S. (2019). Prevalence and management of hypokalemia in peritoneal dialysis patients in Qatar. Int J Nephrol.

[33] Tangjittrong K. (2021). Risk factors for the first episode of peritonitis in continuous ambulatory peritoneal dialysis patients in Pranangklao Hospital. J Med Assoc Thai.

[34] Xu Q., Xu F., Fan L. (2014). Serum potassium levels and its variability in incident peritoneal dialysis patients: associations with mortality. PLOS ONE.

[35] Liu Y., Cheng B.C., Lee W.C. (2016). Serum potassium profile and associated factors in incident peritoneal dialysis patients. Kidney Blood Press Res.

[36] Tatiyanupanwong S., Laohasiriwong W., Chaichaya N., Thinkhamrop B., Thinkhamrop J., Chan-on C. (2020). The effect of hypokalemia on early-onset peritoneal dialysis-related peritonitis. Medico-Legal Update.

[37] Pan J., Xu X., Wang Z., Ma T., Dong J. (2023). Dietary potassium and clinical outcomes among patients on peritoneal dialysis. Nutrients.

[38] Palmer B.F. (2020). Potassium binders for hyperkalemia in chronic kidney disease-diet, renin-angiotensin-aldosterone system inhibitor therapy, and hemodialysis. Mayo Clin Proc.

[39] Song Y., Lobene A.J., Wang Y., Hill Gallant K.M. (2021). The DASH diet and cardiometabolic health and chronic kidney disease: a narrative review of the evidence in East Asian countries. Nutrients.

[40] Carrero J.J., Thomas F., Nagy K. (2018). Global prevalence of protein-energy wasting in kidney disease: a meta-analysis of contemporary observational studies from the International Society of Renal Nutrition and Metabolism. J Ren Nutr.

[41] Kwan B.C.H., Szeto C.C. (2012). Dialysis: hypokalaemia and cardiac risk in peritoneal dialysis patients. Nat Rev Nephrol.

[42] Unwin R.J., Luft F.C., Shirley D.G. (2011). Pathophysiology and management of hypokalemia: a clinical perspective. Nat Rev Nephrol.

[43] Kalantar-Zadeh K., Ikizler T.A., Block G., Avram M.M., Kopple J.D. (2003). Malnutrition-inflammation complex syndrome in dialysis patients: causes and consequences. Am J Kidney Dis.

[44] Kovesdy C.P., Matsushita K., Sang Y. (2018). Serum potassium and adverse outcomes across the range of kidney function: a CKD Prognosis Consortium meta-analysis. Eur Heart J.

[45] Zhang Y., Chen P., Chen J., Wang L., Wei Y., Xu D. (2019). Association of low serum potassium levels and risk for all-cause mortality in patients with chronic kidney disease: a systematic review and meta-analysis. Ther Apher Dial.

[46] Zhang J., Lu X., Li H., Wang S. (2021). Risk factors for mortality in patients undergoing peritoneal dialysis: a systematic review and meta-analysis. Ren Fail.

[47] Wang Q., Bernardini J., Piraino B., Fried L. (2003). Albumin at the start of peritoneal dialysis predicts the development of peritonitis. Am J Kidney Dis.

[48] Nakai K., Saito K., Fujii H., Nishi S. (2017). Impact of hypokalemia on peritonitis in peritoneal dialysis patients: a systematic review. Ren Replace Ther.

[49] Fülöp T., Zsom L., Rodríguez B. (2017). Clinical utility of potassium-sparing diuretics to maintain normal serum potassium in peritoneal dialysis patients. Perit Dial Int.

