# Supplementary material for: Hypokalemia in Peritoneal Dialysis: A Systematic Review and Meta-analysis of Prevalence, Treatment, and Outcomes
Source: Kidney Med. 2024 Oct 18;6(12):100923. doi: 10.1016/j.xkme.2024.100923 (PMC11616037; doi:10.1016/j.xkme.2024.100923)
Supplement: Supplementary File (PDF) — Figures S1-S5; Tables S1-S9. [file mmc1.pdf]

Figure S1. The relationship between hypokalemia and all-cause mortality in patients undergoing peritoneal dialysis stratified by potassium levels. A. Serum potassium levels below 4.0mmol/L, B. Serum potassium levels below 3.5mmol/L, C. Serum potassium levels below 3.0mmol/L.

A. Serum potassium levels below 4.0mmol/L.

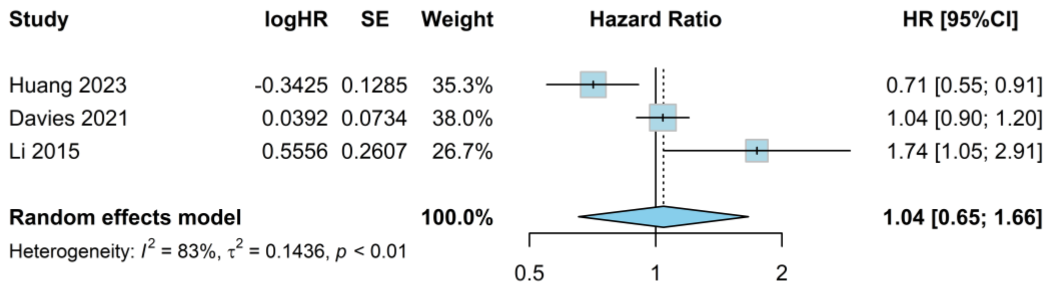

B. Serum potassium levels below 3.5mmol/L.

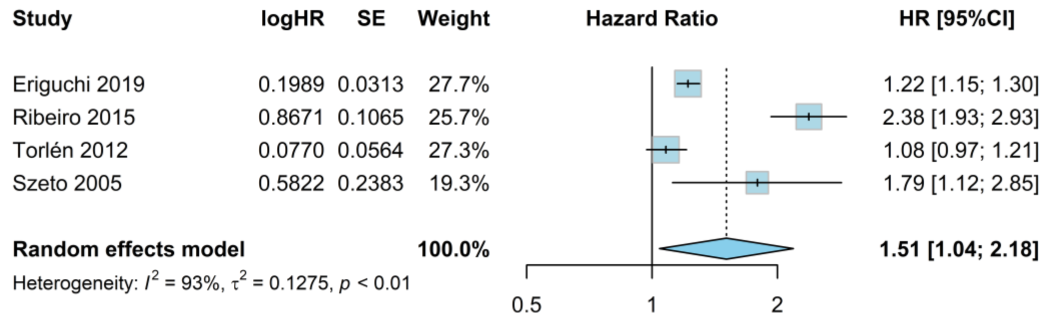

C. Serum potassium levels below 3.0mmol/L.

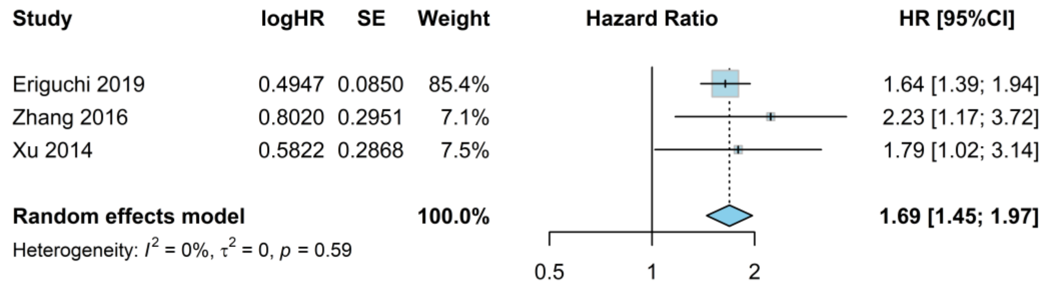

Figure S2. The relationship between hypokalemia and cardiovascular mortality in patients undergoing peritoneal dialysis stratified by potassium levels. A. Serum potassium levels below 4.0mmol/L, B. Serum potassium levels below 3.5mmol/L, C. Serum potassium levels below 3.0mmol/L.

A. Serum potassium levels below 4.0mmol/L.

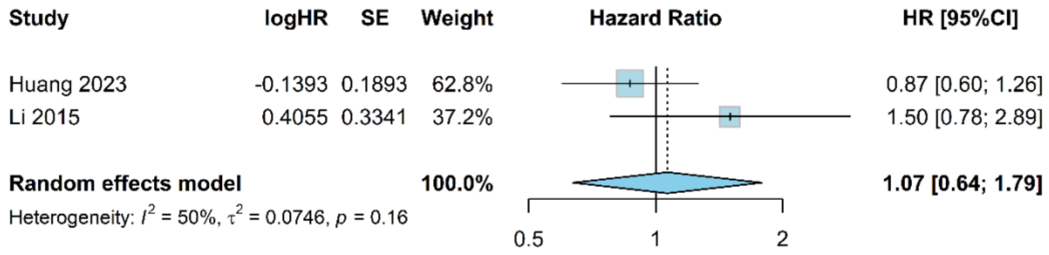

B. Serum potassium levels below 3.5mmol/L.

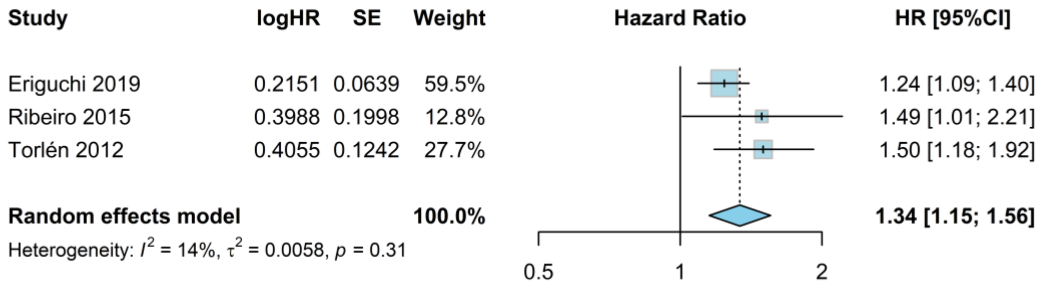

C. Serum potassium levels below 3.0mmol/L.

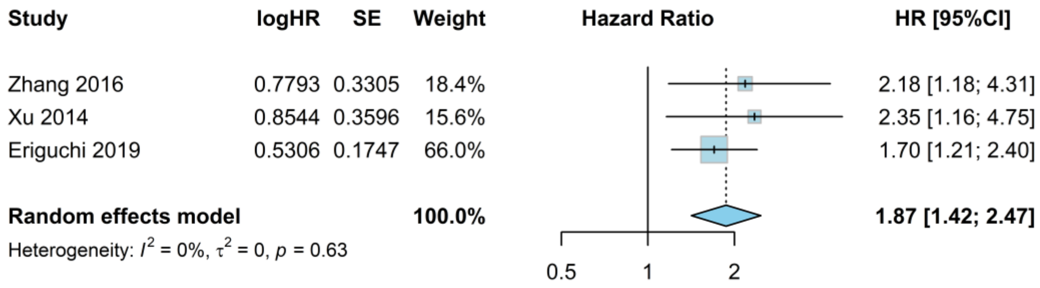

Figure S3. Leave-one-out sensitivity analysis showing the relationship between hypokalemia (as defined by study definition) and all-cause mortality in patients receiving peritoneal dialysis.

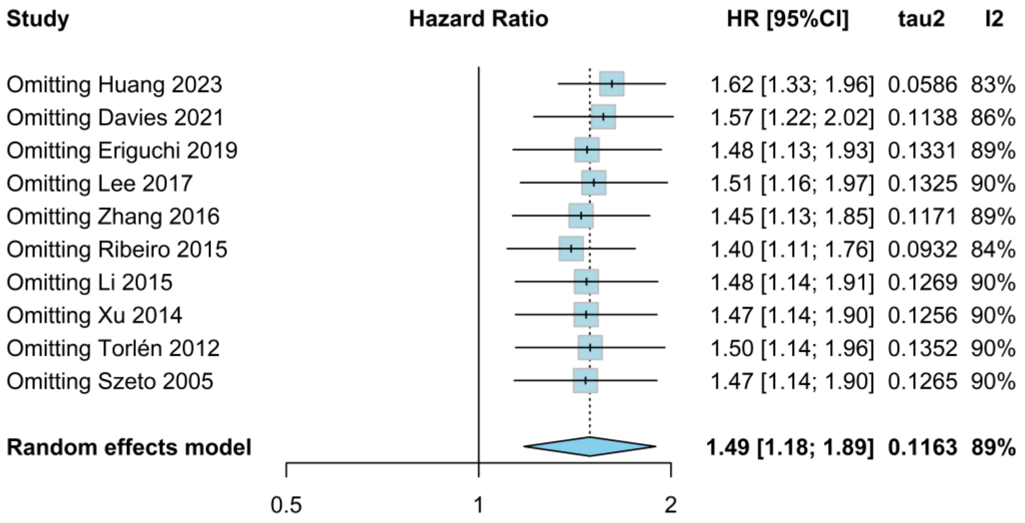

Figure S4. Funnel plot depicting publication bias in included studies investigating the prevalence of hypokalemia (as defined below 3.5mmol/L) (Egger's test *p* value = 0.65).

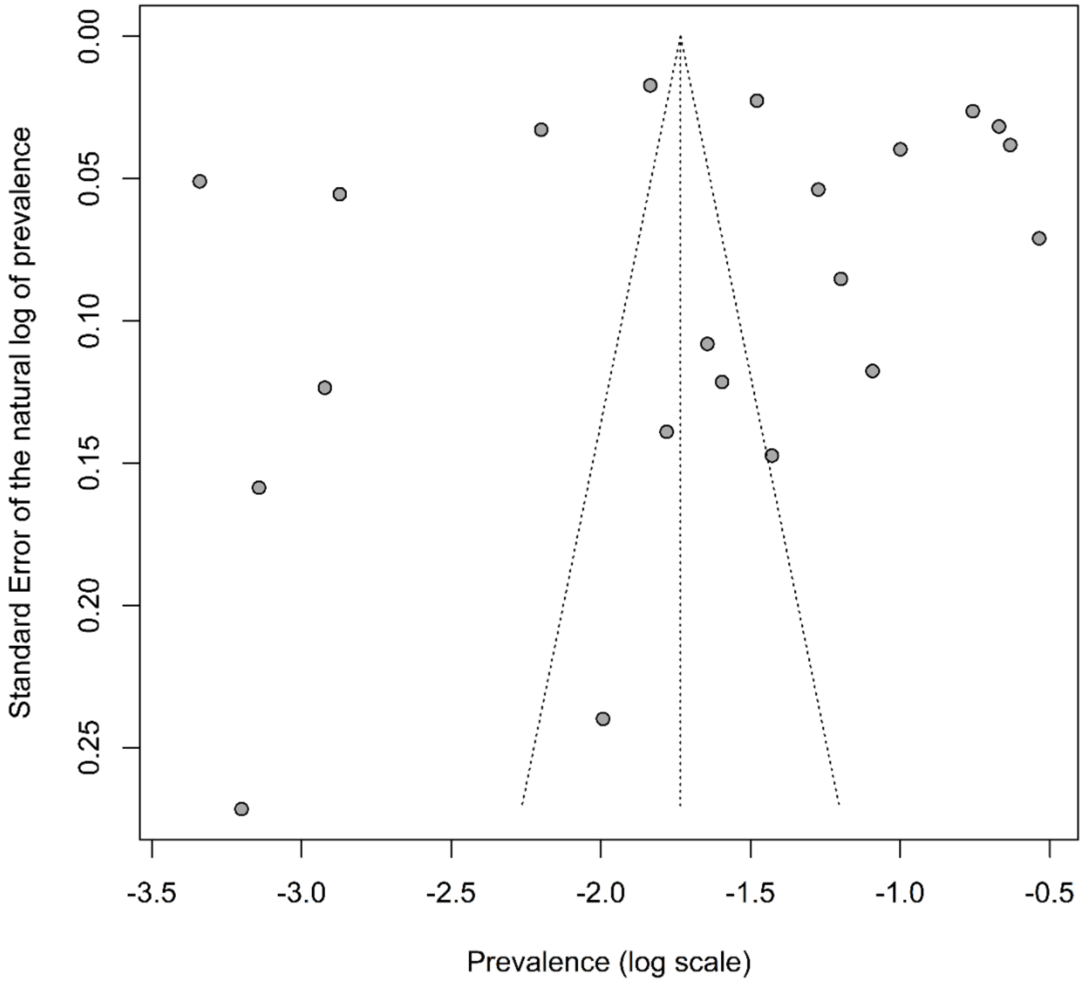

Figure S5. Funnel plot depicting publication bias in included studies investigating the associations between hypokalemia (as defined by study definition) and all-cause mortality (Egger's test *p* value =0.55).

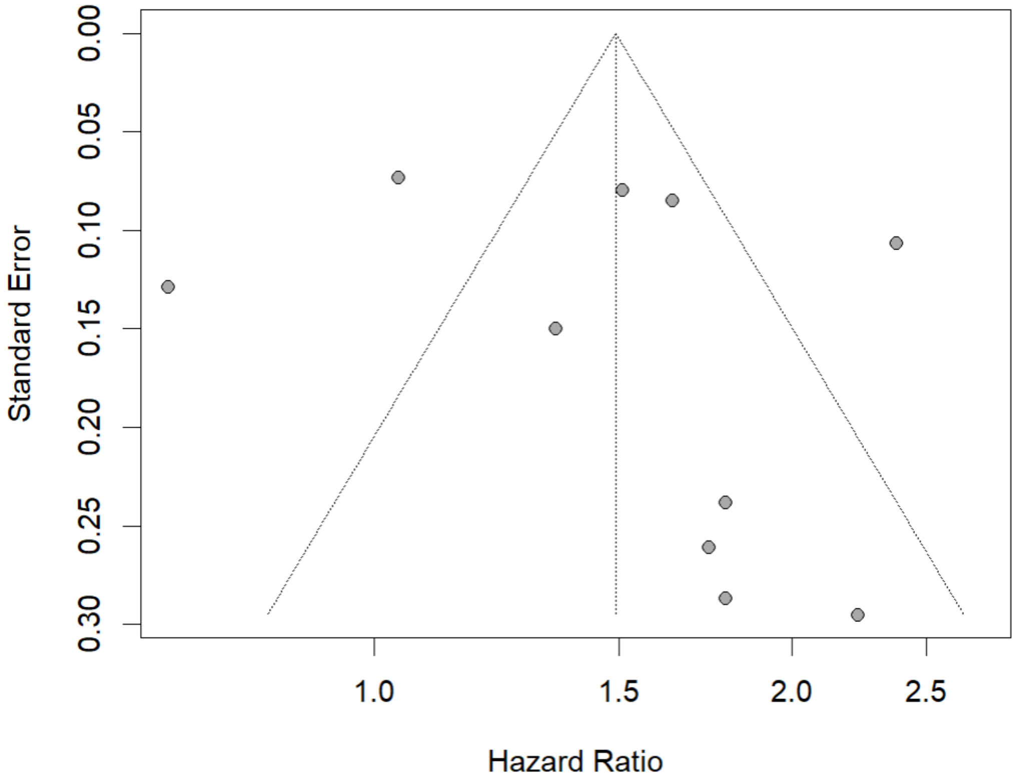

Table S1. PRISMA checklist.

Section and Topic	Item #	Checklist item	Location where item is reported
**TITLE**			
Title	1	Identify the report as a systematic review.	Page 1
**ABSTRACT**			
Abstract	2	See the PRISMA 2020 for Abstracts checklist.	Page 3
**INTRODUCTION**			
Rationale	3	Describe the rationale for the review in the context of existing knowledge.	Page 5
Objectives	4	Provide an explicit statement of the objective(s) or question(s) the review addresses.	Page 5
**METHODS**			
Eligibility criteria	5	Specify the inclusion and exclusion criteria for the review and how studies were grouped for the syntheses.	Page 6
Information sources	6	Specify all databases, registers, websites, organisations, reference lists and other sources searched or consulted to identify studies. Specify the date when each source was last searched or consulted.	Page 6
Search strategy	7	Present the full search strategies for all databases, registers and websites, including any filters and limits used.	Table S1
Selection process	8	Specify the methods used to decide whether a study met the inclusion criteria of the review, including how many reviewers screened each record and each report retrieved, whether they worked independently, and if applicable, details of automation tools used in the process.	Page 6
Data collection process	9	Specify the methods used to collect data from reports, including how many reviewers collected data from each report, whether they worked independently, any processes for obtaining or confirming data from study investigators, and if applicable, details of automation tools used in the process.	Page 6
Data items	10a	List and define all outcomes for which data were sought. Specify whether all results that were compatible with each outcome domain in each study were sought (e.g. for all measures, time points, analyses), and if not, the methods used to decide which results to collect.	Page 7
	10b	List and define all other variables for which data were sought (e.g. participant and intervention characteristics, funding sources). Describe any assumptions made about any missing or unclear information.	Table S3
Study risk of bias assessment	11	Specify the methods used to assess risk of bias in the included studies, including details of the tool(s) used, how many reviewers assessed each study and whether they worked independently, and if applicable, details of automation tools used in the process.	Page 7
Effect measures	12	Specify for each outcome the effect measure(s) (e.g. risk ratio, mean difference) used in the synthesis or presentation of results.	Page 8
Synthesis methods	13a	Describe the processes used to decide which studies were eligible for each synthesis (e.g. tabulating the study intervention characteristics and comparing against the planned groups for each synthesis (item #5)).	Page 8
	13b	Describe any methods required to prepare the data for presentation or synthesis, such as handling of missing summary statistics, or data conversions.	Page 8
	13c	Describe any methods used to tabulate or visually display results of individual studies and syntheses.	Page 8
	13d	Describe any methods used to synthesize results and provide a rationale for the choice(s). If meta-analysis was performed, describe the model(s), method(s) to identify the presence and extent of statistical heterogeneity, and software package(s) used.	Page 7
	13e	Describe any methods used to explore possible causes of heterogeneity among study results (e.g. subgroup analysis, meta-regression).	Page 8
	13f	Describe any sensitivity analyses conducted to assess robustness of the synthesized results.	Page 8
Reporting bias	14	Describe any methods used to assess risk of bias due to missing results in a synthesis (arising from reporting biases).	Page 8

Section and Topic	Item #	Checklist item	Location where item is reported
assessment			
Certainty assessment	15	Describe any methods used to assess certainty (or confidence) in the body of evidence for an outcome.	Page 8
**RESULTS**			
Study selection	16a	Describe the results of the search and selection process, from the number of records identified in the search to the number of studies included in the review, ideally using a flow diagram.	Page 9
	16b	Cite studies that might appear to meet the inclusion criteria, but which were excluded, and explain why they were excluded.	Page 9
Study characteristics	17	Cite each included study and present its characteristics.	Page 9
Risk of bias in studies	18	Present assessments of risk of bias for each included study.	Page 9
Results of individual studies	19	For all outcomes, present, for each study: (a) summary statistics for each group (where appropriate) and (b) an effect estimate and its precision (e.g. confidence/credible interval), ideally using structured tables or plots.	Page 9-10
Results of syntheses	20a	For each synthesis, briefly summarise the characteristics and risk of bias among contributing studies.	Page 9-10
	20b	Present results of all statistical syntheses conducted. If meta-analysis was done, present for each the summary estimate and its precision (e.g. confidence/credible interval) and measures of statistical heterogeneity. If comparing groups, describe the direction of the effect.	Page 9-10
	20c	Present results of all investigations of possible causes of heterogeneity among study results.	Page 10
	20d	Present results of all sensitivity analyses conducted to assess the robustness of the synthesized results.	Page 11
Reporting biases	21	Present assessments of risk of bias due to missing results (arising from reporting biases) for each synthesis assessed.	Page 11
Certainty of evidence	22	Present assessments of certainty (or confidence) in the body of evidence for each outcome assessed.	Page 11
**DISCUSSION**			
Discussion	23a	Provide a general interpretation of the results in the context of other evidence.	Page 12
	23b	Discuss any limitations of the evidence included in the review.	Page 14
	23c	Discuss any limitations of the review processes used.	Page 14
	23d	Discuss implications of the results for practice, policy, and future research.	Page 14
**OTHER INFORMATION**			
Registration and protocol	24a	Provide registration information for the review, including register name and registration number, or state that the review was not registered.	Page 6
	24b	Indicate where the review protocol can be accessed, or state that a protocol was not prepared.	Page 6
	24c	Describe and explain any amendments to information provided at registration or in the protocol.	Page 6
Support	25	Describe sources of financial or non-financial support for the review, and the role of the funders or sponsors in the review.	Page 15
Competing interests	26	Declare any competing interests of review authors.	Page 15
Availability of data, code and	27	Report which of the following are publicly available and where they can be found: template data collection forms; data extracted from included studies; data used for all analyses; analytic code; any other materials used in the review.	Page 16

Section and Topic	Item #	Checklist item	Location where item is reported
other materials			

From: Page MJ, McKenzie JE, Bossuyt PM, Boutron I, Hoffmann TC, Mulrow CD, et al. The PRISMA 2020 statement: an updated guideline for reporting systematic reviews. BMJ 2021;372:n71. doi: 10.1136/bmj.n71

For more information, visit: http://www.prisma-statement.org/

Table S2. Search strategy.

**MEDLINE**
#1Potassium[MeSH Terms] OR Potassium[Title/Abstract]<br>#2 hypokalemia[MeSH Terms] OR hypokalemia[Title/Abstract] OR Hypokalemias[Title/Abstract] OR Hypopotassemia[Title/Abstract] OR Hypopotassemias[Title/Abstract]<br>#3 #1 OR #2<br>#4 Peritoneal Dialysis[MeSH Terms] OR "Peritoneal Dialysis"[Title/Abstract] OR Peritoneal Dialyses[Title/Abstract] OR "Dialyses, Peritoneal"[Title/Abstract] OR "Dialysis, Peritoneal"[Title/Abstract] OR "Peritoneal Dialyses"[Title/Abstract]<br>#5 Peritoneal Dialysis, Continuous Ambulatory[MeSH Terms] OR "Continuous Ambulatory Peritoneal Dialysis"[Title/Abstract]<br>#6 #4 OR #5<br>#7 #3 AND #6
**Web of science**
#1 TS=Potassium<br>#2 TS=hypokalemia<br>#3 TS=Hypokalemias<br>#4 TS=Hypopotassemias<br>#5 #1 OR #2 OR #3 OR #4<br>#6 TS=peritoneal dialysis<br>#7 TS=Peritoneal Dialyses<br>#8 TS=Peritoneal Dialysis, Continuous Ambulatory<br>#9 #6 OR #7 OR #8<br>#10 #5 AND #9
**Embase**
#1 'potassium'/exp OR potassium<br>#2 'hypokalemia'/exp OR hypokalemia<br>#3 hypokalemias<br>#4 hypopotassemias<br>#5 #1 OR #2 OR #3 OR #4<br>#6 'peritoneal dialysis'/exp OR 'peritoneal dialysis'<br>#7 'peritoneal dialyses'<br>#8 'continuous ambulatory peritoneal dialysis'/exp OR 'continuous ambulatory peritoneal dialysis'<br>#9 #6 OR #7 OR #8<br>#10 #5 AND #9

Table S3. Summary of items extracted.

Characteristics of research	The title of the study, first author name, year of publication, journal, country/region of study, study design, and statistical analysis approaches (including adjustment for confounding variables).
Characteristics of study population	Total sample size, age, sex, and duration of follow up
Definition/threshold of hypokalemia	Threshold, duration, and definition of hypokalemia.
Outcomes of interest	• The number of individuals with hypokalemia.<br>• Prevalence of hypokalemia, which was defined as the proportion of prevalent PD patients with hypokalemia as the numerator and total study population as the denominator, multiplied by 100 to express it as a percentage. Where possible, this method was also used to determine hypokalemia prevalence for all subgroups assessed.<br>• Outcomes (all-cause mortality, cardiovascular mortality, infection-related mortality, and PD-related peritonitis) were extracted as the most adjusted hazard ratios (HR) or odds ratios (OR) and the 95% confidence interval (95% CI). Cardiovascular mortality was defined as sudden death or death attributed to acute myocardial infarction, pericarditis, atherosclerotic heart disease, cardiomyopathy, cardiac arrhythmia, cardiac arrest, valvular heart disease, pulmonary edema, or congestive heart failure. Infection-related mortality was defined as death primarily caused by an acute infection (such as pneumonia, sepsis, urinary tract infections, gastrointestinal infections).<br>• If data was reported in figures and could not be located in text, supplemental material or through author contact, we extracted the data directly from figures using Webplot designer (https://automeris.io/webplotdigitizer/).
Pharmacological or dietary interventions	Duration of potassium intervention phase, and type and quantity of the potassium supplements.

Table S4. Summary of 15 studies investigating exposure to hypokalemia (mmol/L) versus adverse outcomes in patients receiving PD.

**Study (Year)**	**Exp.**	**Ref.**	**Outcomes and study estimates HR/OR (95% CI)**				
				All-cause mortality	Cardiovascular mortality	Infection-related mortality	PD-associated peritonitis
**Huang, 2023**	<4.0	>=4.0	HR	0.71 (0.55-0.91)	0.87 (0.60-1.26)	NA	NA
**Huo, 2022**	<3.5	>=3.5	HR	NA	NA	NA	1.53 (1.14-2.06)
**Davies, 2021**	<3.5<br><4.0	4.0-4.4	HR	 1.04 (0.90-1.20)	NA	NA	1.15 (0.96-1.37)
**Tangjittrong, 2021**	<3.5	>=3.5	OR	NA	NA	NA	0.84 (0.56-1.26)
**Liu, 2021**	<3.0<br>3.0-3.5<br><3.5	>=3.5	HR	NA	NA	NA	2.10 (1.40-3.13)<br>1.07 (0.71-1.61)<br>1.44 (1.01-2.04)
**Tatiyanupanwong, 2020**	<3.5	>=3.5	OR	NA	NA	NA	2.36 (1.42-3.94)
**Eriguchi, 2019**	<2.5<br><3.0<br><3.5	>=4.0	HR	2.20 (1.66-2.93)<br>1.64 (1.39-1.94)<br>1.22 (1.15-1.30)	2.36 (1.31-4.24)<br>1.70 (1.21-2.40)<br>1.24 (1.09-1.40)	NA	NA
**Lee, 2017**	<4.5	>=4.5	HR	1.35 (1.00-1.80)	NA	NA	NA
**Zhang, 2016**	<3.0<br>3.0-4.0	4.0-5.0	HR	2.23 (1.17-3.72)<br>1.35 (0.89-1.81)	2.18 (1.18-4.31)<br>1.45 (0.92-1.99)	NA	NA
**Li, 2015**	<4.0	>=4.0	HR	1.74 (1.05-2.91)	1.50 (0.78-2.89)	NA	NA
**Ribeiro, 2015**	<3.5<br>3.5-4.0	4.0-4.5	HR	2.38 (1.93-2.93)	1.49 (1.01-2.21)<br>1.29 (1.01-1.66)	 <br>1.93 (1.38-2.70)	NA
**Fan, 2014**	<3.5	>=3.5	HR	NA	NA	NA	1.15 (0.91-1.47)
**Xu, 2014**	<3.0<br>3.0-3.5<br>3.5-4.0	4.0-4.5	HR	1.79 (1.02-3.14)<br>1.15 (0.72-1.86)<br>1.31 (0.82-2.08)	2.35 (1.16-4.75)<br>1.25 (0.66-2.37)<br>1.24 (0.65-2.33)	NA	NA
**Torlén,2012**	<3.5<br>3.5-4.0	4.0-4.5	HR	1.51 (1.29-1.76)<br>1.12 (1.03-1.21)	1.50 (1.18-1.92)<br>1.04 (0.91-1.18)	1.85 (1.35-2.55)<br>1.15 (0.96-1.38)	NA
**Szeto, 2005**	<3.5	>=3.5	HR	1.79 (1.12-2.85)	NA	NA	NA

The confounders adjusted in each of the included studies were summarized in Supplementary Table S4.

Abbreviation: Exp.: Exposure, Ref.: Reference, OR: odds ratio, HR: hazard ratio, NA: not available, PD: Peritoneal dialysis.

Table S5. Potential confounders were adjusted for in the included studies.

Study	Adjusted potential confounders
Huang, 2023	Age, gender, DM, CVD history, dialysis duration, uric acid, bicarbonate, hemoglobin, cholesterol, triglyceride, LDL-c, phosphorus, calcium, iPTH and albumin.
Pan, 2023	Age, gender, BMI, educational level, DM, CVD, diastolic blood pressure, albumin, serum creatinine, serum potassium, serum phosphorus, total cholesterol, peritoneal dialysis KT/V, residual kidney KT/V, and Hs-CRP
Huo, 2022	Age, gender, BMI, education level, smoking, diabetes, CVD history, RASi medications, RRF loss, baseline potassium, the mean values of dialysate GLUC, total weekly Kt/V score, serum albumin, serum creatinine and serum phosphorus.
Davies, 2021	Age, sex, kidney failure vintage, CAD, cancer, other CVD, cerebrovascular disease, CHF, DM, gastrointestinal bleeding, hypertension, lung disease, neurologic disease, psychiatric disorder, peripheral vascular disease, recurrent cellulitis/gangrene phosphorous, bicarbonate, creatinine, albumin, caregiver involvement, APD, icodextrin, PD solution type, 24 h urine volume, loop diuretic, ACEi or ARB, peritoneal Kt/V urea.
Tangjittrong, 2021	Age, gender, education level, DM, serum hemoglobin, and serum albumin level.
Liu, 2021	Age, sex, BMI, the mean values of serum albumin, hemoglobin, and Kt/V during the first 12m after dialysis initiation.
Tatiyanupanwong, 2020	Age, gender, DM, albumin, eGFR, total lymphocyte count, BMI, calcium, phosphate, BUN, and sodium.
Eriguchi, 2019	Age, sex, race, DM, insurance, hypertension, CVD, CHF, other CVD, seven surrogates of nutritional and inflammatory status, BMI, hemoglobin, and serum albumin, creatinine, calcium, phosphorus, and bicarbonate.
Lee, 2017	Age, sex, modified CCI, BMI, subjective global assessment, albumin, alkaline phosphatase, Uric acid, phosphorous, and calcium.
Zhang, 2016	Age, sex, BMI, etiology of kidney failure, DM, duration of dialysis, total Kt/v urea and CCI.
Li, 2015	Age, gender, BMI, DM, CCI, hemoglobin, serum albumin, hs-CRP, and time-averaged serum potassium.
Ribeiro, 2015	Age, gender, BMI, center experience, Davies score, DM, family income, literacy, PD modality, race, previous HD, duration of pre-dialysis care and year of initiation of PD.
Xu, 2014	Age, gender, BMI, DM, CCI, hemoglobin, serum albumin, hs-CRP, and PDV/BSA
Fan, 2014	Age, Gender, educational level, Albumin, hemoglobin and potassium.
Torlén,2012	Age, sex, race and/or ethnicity, DM, dialysis vintage, primary insurance, marital status, CVD, CHF, cerebrovascular disease, other CVD, peripheral vascular disease, COPD, cancer, current smoking, BMI, serum albumin, total iron binding capacity, ferritin, creatinine, calcium, phosphorus, parathyroid hormone, alkaline phosphatase, hemoglobin, WBC count, and percentage
Szeto, 2005	Age, time on dialysis, DM, CCI, Subjective Global Assessment, serum albumin, serum potassium, anthropometric LBM, total Kt/V, nPNA,

	FEBM, and residual GFR.

Abbreviation: DM: diabetes mellitus, CVD: Cardiovascular disease, iPTH: Intact parathyroid hormone; LDL-C: Low-density lipoprotein cholesterol, BMI: body mass index, RASi: renin-angiotensin system inhibitor, RRF: residual renal function, GLUC: dialysate glucose concentration, CAD: Coronary artery disease, CHF: Congestive heart failure, ACEi: angiotensin-converting enzyme inhibitor, APD: automated peritoneal dialysis, ARB: angiotensin II receptor blockers, eGFR: estimated glomerular filtration rate, BUN: blood urea nitrogen, CCI: Charlson comorbidity index, HD: hemodialysis, Hs-CRP: high-sensitivity C-reactive protein, PDV/BSA: peritoneal dialysis volume per unit of body surface area, COPD: chronic obstructive pulmonary disease, WBC: white blood cell, LBM: anthropometric lean body mass, nPNA: normalized protein nitrogen appearance, FEBM: fat-free edema-free body mass.

Table S6. Quality assessment using the Newcastle-Ottawa Quality Assessment Scale for cohort study.

Study	Selection				Comparability	Outcome			
	Representativeness of the exposed cohort	Selection of the non-exposed cohort	Ascertainment of exposure	Demonstration that outcome of interest was not present at start of study	Comparability of cohorts on the basis of the design or analysis	Assessment of outcome	Was follow-up long enough for outcomes to occur	Adequacy of follow up of cohorts	Total Star ★
Huang 2023	★	★	☆	★	★	★	★	★	7
Pan 2023	★	★	★	★	★	★	★	★	8
Huo 2022	★	★	★	★	★	★	★	★	8
Tangjittrong 2021	★	★	☆	★	★	★	☆	☆	5
Liu 2021	★	★	★	★	★	★	★	★	8
Davies 2021	★	★	★	★	★	★	★	★	8
Tatiyanupanwong 2020	★	★	★	★	★	★	☆	☆	6
Eriguchi 2019	★	★	★	★	★	★	★	★	8
Lee 2017	★	★	★	★	★	★	★	★	8
Zhang 2016	★	★	★	★	★	★	★	★	8
Ribeiro 2015	★	★	★	★	★	★	★	★	8
Li 2015	★	★	★	★	★	★	★	★	8
Xu 2014	★	★	★	★	★	★	★	★	8
Fan 2014	★	★	★	★	-	★	★	★	7
Torlén 2012	★	★	★	★	★	★	★	★	8
Liawnoraset 2011	★	★	☆	★	-	★	★	★	6
Yi 2009	★	★	★	★	-	★	★	★	7
Chuang 2009	★	★	☆	★	-	★	★	★	6
Szeto 2005	★	★	★	★	★	★	★	★	8

★Indicate low risk of bias, ☆ Indicate high risk of bias, - indicate unclear risk of bias.

Table S7. Quality assessment using Cochrane Collaboration's tool for randomized controlled trials.

Study	Random sequence generation	Allocation concealment	Blinding of participants and personnel	Blinding of outcome assessment	Incomplete outcome data	Selective reporting	Other bias
Pichitporn 2022	low risk	unclear	unclear	low risk	low risk	low risk	unclear

Table S8. GRADE table summarizing the quality of the evidence for each outcome in the meta-analysis.

Certainty assessment							Effect	Certainty
	**No. of studies**	**Study design**	**Risk of bias**	**Inconsistency**	**Indirectness**	**Imprecision**	**% /HR (95% CI)**	
**Prevalence of hypokalemia**								
Potassium level below 4.0	7	observational studies	not serious	serious[a]	serious[b]	serious[c]	37.9 (27.2-52.7)	⊕○○○ VERY LOW
Potassium level below 3.5	21	observational studies	not serious	serious[a]	serious[b]	serious[c]	17.7 (12.0-25.9)	⊕○○○ VERY LOW
Potassium level below 3.0	8	observational studies	not serious	serious[a]	serious[b]	serious[c]	4.4 (1.9-10.2)	⊕○○○ VERY LOW
**Outcomes**								
All-cause mortality	10	observational studies	not serious	serious[a]	not serious	not serious	1.49 (1.18-1.89)	⊕⊕○○ LOW
Cardiovascular mortality	7	observational studies	not serious	not serious	not serious	not serious	1.50 (1.19-1.88)	⊕⊕⊕○ MODERATE
PD-associated peritonitis	6	observational studies	not serious	serious[d]	not serious	not serious	1.53 (1.23-1.88)	⊕⊕○○ LOW

Abbreviations: HR: hazard ratio, CI: Confidence interval, PD: peritoneal dialysis.

a. High heterogeneity

b. The definition of hypokalemia varied across the most of included studies

c. Very wide confidence intervals

d. Moderate heterogeneity

Table S9. Quality Assessment using the Joanna Briggs Institute Critical Appraisal Checklist for Studies Reporting Prevalence Data.

Study	1. Sampling Frame	2. Appropriate Sampling	3. Sample Size	4. Subject and Setting	5. Analysis Coverage	6. Valid Measures	7. Standardized Measures	8. Statistical Analysis	9. Response Rate	Total
**Goncalves 2020**	Y	Y	Y	Y	Y	Y	Y	N	Y	8
**Hamad 2019**	Y	Y	Y	Y	Y	Y	Y	N	Y	8
**Liu 2016**	Y	Y	Y	Y	Y	Y	Y	N	N	8
**Vavruk 2012**	Y	Y	Y	Y	unclear	Y	Y	N	Y	6

Table S10. Meta-regression analyses on the association between hypokalemia and adverse outcomes.

Hypokalemia vs non-hypokalemia	No. of studies	Empirical Bayes meta-regression pooled HR (95% CI)	*P* value	I^2 (%)
**All-cause mortality**				
**Sample size ≥1000 vs <1000**	10	0.72 (0.44-1.19)	0.20	88.9
**Non-Asia vs Asia**	9	1.26 (0.76-2.09)	0.37	85.6
**Cardiovascular mortality**				
**Sample size ≥1000 vs <1000**	7	0.70 (0.42-1.16)	0.16	47.9
**Non-Asia vs Asia**	7	1.08 (0.66-1.78)	0.75	55.7
**PD-related peritonitis**				
**Sample size ≥1000 vs <1000**	5	0.83 (0.52-1.33)	0.45	55.8
**Non-Asia vs Asia***	4	NA	NA	NA

*All included studies were from Asia. HR: Hazard ratio; CI: confidence interval.

18
